# Expanded Indication for Magnetic Sphincter Augmentation: Outcomes in Weakly Acidic Reflux Compared to Standard GERD Patients

**DOI:** 10.1007/s11605-021-05152-5

**Published:** 2021-09-29

**Authors:** Milena Nikolic, Aleksa Matic, Joy Feka, Lisa Gensthaler, Ivan Kristo, Bogdan Osmokrovic, Franz M. Riegler, Berta O. Mosleh, Sebastian F. Schoppmann

**Affiliations:** 1 Department of Surgery, Medical University of Vienna, Waehringer Guertel 18-20, 1090 Vienna, Austria; 2 Department of Trauma Surgery, Krankenhaus Oberwart, Dornburggasse 80, 7400 Oberwart, Austria; 3 Reflux Ordination, Mariannengasse 10/4/9, 1090 Vienna, Austria

**Keywords:** GERD, Magnetic sphincter augmentation, Weakly acid, Hiatal hernia

## Abstract

**Background:**

Magnetic sphincter augmentation (MSA) is a modern surgical anti-reflux technique with proven efficacy and low postoperative morbidity in patients with acidic reflux. The aim of this retrospective review study was to evaluate the symptomatic outcome of MSA in patients with weakly acidic reflux.

**Methods:**

From a prospectively collected clinical database, comprising all 327 patients that underwent MSA at our institution, a total of 67 patients with preoperative weakly acidic reflux measured in the 24-h impedance-pH-metry were identified. Postoperative gastrointestinal symptoms, proton pump inhibitor intake (PPI), GERD Health-Related Quality-of-Life (GERD-HRQL), alimentary satisfaction (AS), and patients’ overall satisfaction were evaluated within highly standardized follow-up appointments. Furthermore, outcome of these patients was compared to the postoperative outcome of a comparable group of patients with a preoperative acidic reflux.

**Results:**

At a median follow-up of 24 months, none of the patients with weakly acidic reflux presented with persistent dysphagia, or underwent endoscopic dilatation or reoperation. The postoperative GERD-HRQL score was significantly reduced (2 vs. 20; *p* = 0.001) and the median AS was 9/10. Preoperative daily heartburn, regurgitations, and respiratory complaints were improved in 95%, 95%, and 96% of patients, respectively. A total of 10% of the patients continued to use PPIs postoperatively. No significant difference was observed in terms of postoperative outcome or quality of life when comparing weakly acidic reflux patients with those diagnosed with preoperative acidic reflux.

**Conclusion:**

Magnetic sphincter augmentation significantly improves GERD-related symptoms and quality of life in patients with weakly acidic reflux with very low postoperative morbidity.

## Introduction

Gastroesophageal reflux disease (GERD) is one of the most common upper gastrointestinal (GI) tract disorders with a worldwide high prevalence and yearly increasing incidence rates.^1–3^ Patients not only suffer from a variety of burdensome symptoms that lead to a reduced quality of life, but also are at an increased risk of developing Barrett’s esophagus (BE) and esophageal adenocarcinoma.^4–9^ The combination of clinical evaluation, upper GI endoscopy, and esophageal function testing, including high-resolution manometry as well as 24-h impedance-pH-metry, is applied to diagnose GERD.^10,11^ Depending on the measured pH in the esophagus, GERD can be divided into acid (pH < 4) or weakly acidic or non-acidic (pH ≥ 4) reflux, both of which cause GERD symptoms and most commonly occur together.^7,12–15^ First-line treatment consists of lifestyle changes and pharmacotherapy with proton pump inhibitors (PPIs).^16^ Suppressing acid production and increasing the pH of the gastric refluxate PPIs effectively eliminate symptoms in approximately 60% of GERD patients. However, medical treatment does not improve the dysfunctional lower esophageal sphincter (LES) and still allows for non-acidic reflux to occur.^17^ Furthermore, not only has mixed acidic and bile reflux been associated with the most severe mucosal injury and deterioration of esophageal function,^18^ but also medical treatment of the acid component has shown to provide only short-term symptom control in patients and not long-term protection of developing BE. The biliary component by itself can cause BE and its degeneration to adenocarcinoma.^19,20^

The gold standard of anti-reflux surgery—the laparoscopic fundoplication (LF) —was shown to have long-term efficacy and safety.^21–26^ Nevertheless, GERD surgery rates have been decreasing in the third millennium, likely due to fear of possible adverse effects, such as dysphagia and gas bloat syndrome.^27–30^ Magnetic sphincter augmentation (MSA) represents an alternative, less invasive surgical option, developed in an effort to decrease side effects associated with LF, while still achieving effective symptom control.^17,22–24,31–33^ The MSA device (LINX® Reflux Management System; Torax Medical, Maple Grove, MN) consists of magnetic beads connected by a flexible titanium ring, which is placed around the LES, meaning to enhance its barrier function and prevent reflux but allow for physiological bolus transport.^34^ Studies have shown the LINX® implantation not only to be as safe and effective as the Nissen fundoplication, but also superior in terms of postoperative gas bloating and increased ability to belch/vomit.^17,31–33,35,36^ Due to the novelty of this procedure, long-term outcome studies of more than 5 years are still needed. Furthermore, one of the questions still unanswered is whether MSA has a comparably good outcome in patients suffering from weakly acidic or non-acidic reflux. Non-acidic reflux is known to cause PPI refractory symptoms in the absence of acid exposure, which makes conservative treatment challenging and underlines the importance of surgical anti-reflux therapy.

The aim of this study was to evaluate the long-term postoperative outcomes of patients with preoperative weakly acidic/biliary reflux undergoing MSA in a high-volume specialized reflux center.

## Methods

### Preoperative Assessment

All patients received a standardized interview, clinical examination, an upper GI endoscopy, a video esophagogram, and esophageal functioning tests consistent of a high-resolution manometry and a 24-h impedance-pH-metry. Patients with the following characteristics were included in our study:

1. Acidic pH < 4 percentage time > 4.2% in the 24-h impedance-pH-metry and/or
2. Increased total reflux episodes > 40 in the 24-h impedance-pH-metry and/or
3. Typical symptoms responsive to PPIs.

Hiatal hernias (HH) were diagnosed with high precision using both upper GI endoscopy and high-resolution manometry. During EGD, a HH was considered if the level of the rise of the endoscopically visible rugal folds dislocated ≥ 1.0 cm above the level of diaphragmatic impression. Weakly acidic reflux was diagnosed in the 24-h impedance-pH-metry by a reduced total acidic (pH < 4) percentage time (< 4.2%) and an increased number of total reflux episodes (> 40). All patients needed to discontinue their PPI therapy 14 days prior undergoing the 24-h impedance-pH-metry.

### Surgery

All procedures were performed by the same specialized upper gastrointestinal surgical team. The surgical approach was laparoscopic in all cases. All procedures were standardized regarding the surgeon’s and patient’s positions (anti-Trendelenburg), trocar sites, and instruments used. A hiatoplasty was performed in 81% of patients. Since the year 2014, hiatoplasty was performed in all patients in principle. These procedures were conducted by hiatal dissections and crural closures with 2–5 stitches using non-absorbable sutures. All cases were performed without the use of an esophageal bougie.

### Magnetic Sphincter Augmentation

MSA was performed as previously described:^17^ briefly after the mobilization of the esophagogastric junction and identifying the vagal nerve and excluding it, the adequate ring size was measured with the sizing tool and the magnetic ring was wrapped around the lower end of the lower esophageal sphincter.

### Sizing of the Device

The sizing tool was placed around the esophagus without applying any tension or compression. It was then closed until it popped off. To make sure that the esophagus was not squeezed, the sizing tool was wiggled. If measurement yielded 10 to 12 beads, then we added 3 beads. If measurement yielded 13, we added 3 or 2, depending when squeezing by the sizing tool occurred*.* If measurement yielded 14 or 15, we added 2, if 17 beads were available. If measurement yielded 16, we used this size if no squeezing by the sizing tool occurred*.* If measurement yielded 16 and squeezing by the sizing tool occurred, the procedure would not be performed.

### Postoperative Care

Postoperatively, all patients received an unrestricted diet, putting an emphasis on regular intake of foods every 2 h, to avoid the development of dysphagia due to formation of scar tissue surrounding the device.

On the first postoperative day, a contrast swallow with iopamidol was performed in all patients. When showing no abnormality, patients were discharged on the first postoperative day per hospital protocol.

### Postoperative Assessment

The median follow-up time was 24 months (IQR, 10 – 41). Long-term follow-up was performed by the same physician using a standardized interview that assessed postoperative gastrointestinal symptoms, proton pump inhibitor intake, and GERD Health-related Quality-of-Life score (GERD-HRQL). The frequency and severity of postoperative dysphagia were assessed using the classification of Saeed et al., where the ability to swallow can be scored from 0 to 5, where 0 implies the inability to swallow and 5 indicates normal swallowing.^37^

Adverse effects such as complications, hospital readmission, emergency surgery, or elective re-operation were documented. Patients with recurrent symptoms received upper GI endoscopy as well as esophageal functioning tests in selected patients.

Postoperative outcome including symptom relief, PPI intake, dysphagia rates, and quality of life was additionally compared between patients with acidic reflux and those with weakly acidic reflux.

### Statistical Analysis

Statistical analysis was performed using SPSS® statistics 20.0 (IBM, Armonk, NY). The data was described using median (interquartile range) or mean (range). Statistical analysis appropriate for non-parametric data was used. Categorical variables were assessed using the Fisher exact test and continuous data using the Wilcoxon rank test as appropriate. Statistical significance was defined as a *p*-value < 0.05.

This study (2293/2017) was approved by the Institutional Review Board of the Medical University of Vienna, Austria. Methods were carried out in accordance with relevant guidelines and regulations.

## Results

**Fig. 1:**
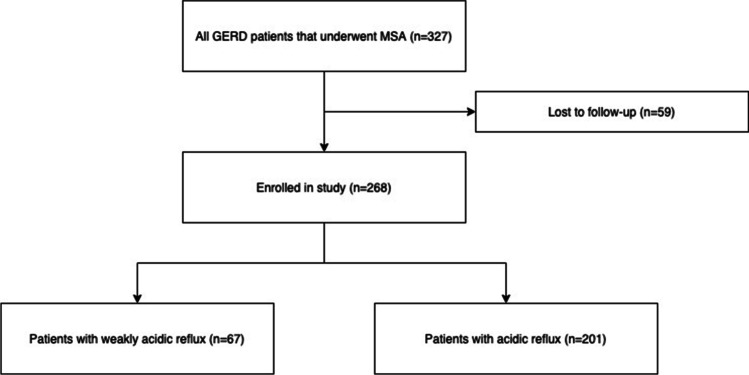
Study flowchart showing all eligible and included patients

A total of 327 patients underwent MSA for chronic gastroesophageal reflux disease in a period of 8 years (2012–2020) in our specialized upper gastrointestinal surgery center. Fifty-nine patients were lost to follow-up, leaving a total number of 268 (100%) patients in our study. Finally, a total of 67 (31 female and 36 male) out of 268 of the individuals were found to have weakly acidic reflux, while 201 (64 female and 137 male) out of 268 individuals revealed acidic reflux in the preoperative 24-h impedance-pH-metry. A study flowchart is shown in Fig. 1.

### Weakly Acidic Reflux Patients (*n* = 67)

The median age was 44 years (IQR, 19, range 23 – 67) and the median preoperative BMI was 25 (IQR, 4.1). Demographics and preoperative findings are shown in Table 1.

**Table 1:** Demographic data and results of preoperative diagnostics of patients with weakly acidic and acidic reflux

	Weakly acidic reflux patients	Acidic reflux patients	
Total number	*n* = 67 (25%)	*n* = 201 (75%)	
Sex (M vs. F)	36 (54%) vs. 31 (46%)	137 (68%) vs. 64 (32%)	*p* = 0.03
Median age (IQR)	44 (19)	51 (21)	*p* = 0.09
Median BMI (IQR)	25 (4.1)	25.5 (5.5)	*p* = 0.122
HH present	57 (85%)	174 (87%)	*p* = 0.584
HH > 3 cm	19 (28%)	43 (21%)	*p* = 0.409
Median total pH < 4% (IQR)	2.2 (1.125) %	7.5 (10.6) %	*p* = 0.00
Median total reflux episodes (IQR)	68 (31.75)	60 (44.5)	*p* = 0.292
Median LES resting pressure (IQR)	20 (16.45) mmHg	16.9 (13.725) mmHg	*p* = 0.059
Median IRP (IQR)	9 (7.75)	9 (6.5)	*p* = 0.465
Presence of IEM	6 (9%)	12 (6%)	*p* = 0.403
Presence of BE	7 (10%)	22 (11%)	*p* = 0.801
Use of PPIs	60 (90%)	175 (87%)	*p* = 0.507

Data were obtained and statistics applied, as described in “Methods” Abbreviations: *HH* hiatal hernia, *LES* lower esophageal sphincter, *IRP* integrated relaxation pressure, *IEM* ineffective esophageal motility, *BE* Barrett’s esophagus

### Preoperative Symptoms

The three most common typical and atypical GERD-associated preoperative symptoms in our patients were heartburn (*n* = 60/67, 90%), regurgitations (*n* = 31/67, 46%), and respiratory symptoms (*n* = 22/67, 32%). A total of 60 out of 67 (90%) of the patients reported the use of PPIs prior to surgery.

### Surgery

The median OR time was 30 min (range, 9 – 52). The surgical approach was laparoscopic in all patients. No perioperative complications were seen. The median MSA device size implanted was 14 (range, 12 – 16). Fifty-two out of 67 (81%) individuals received additional crural closure. The median hospital stay was 1 day (IQR, 1).

### Postoperative Symptom Control

**Fig. 2:**
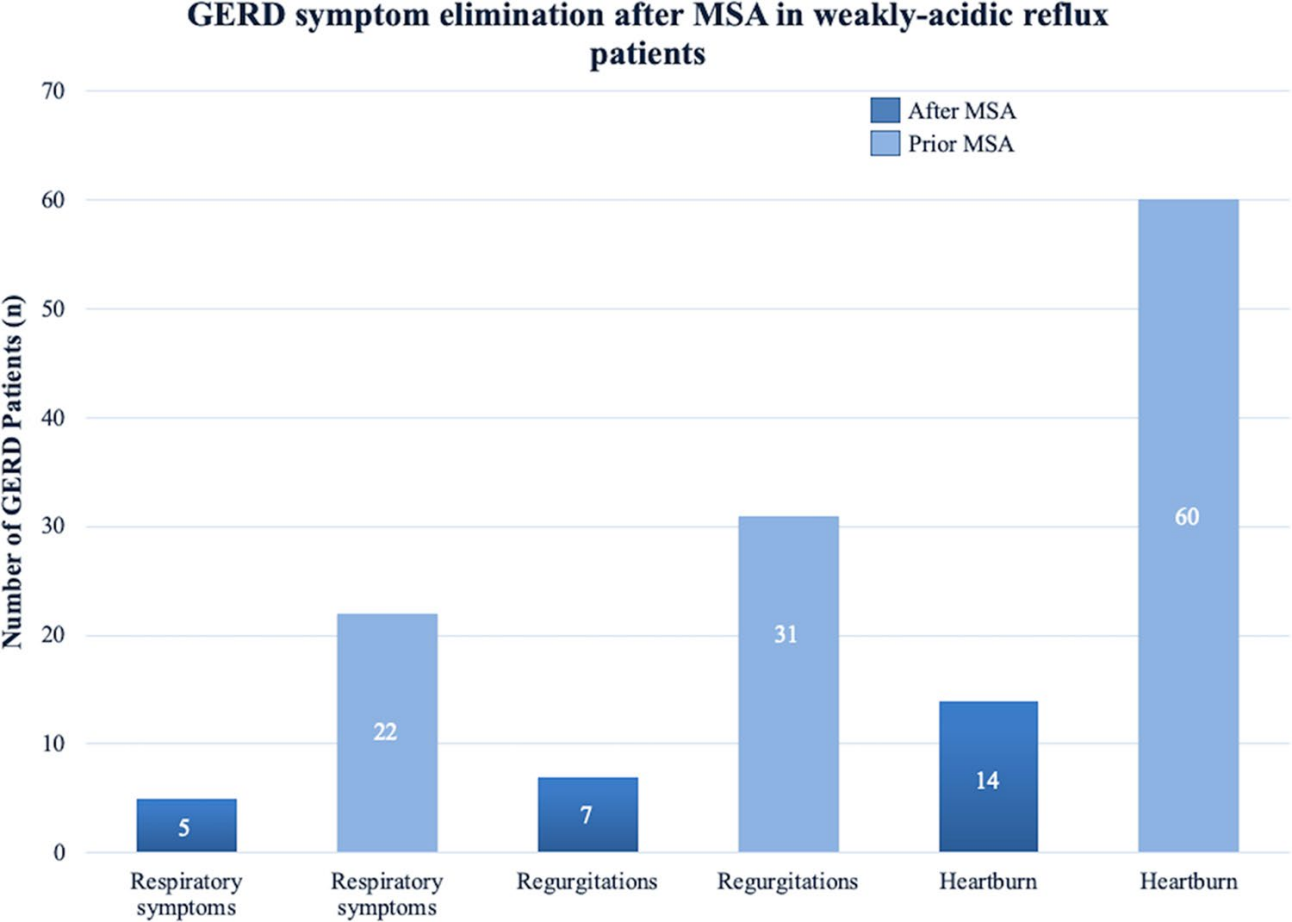
Comparison of GERD-related symptoms before and after MSA in weakly acidic reflux patients

The median follow-up time was 24 months (IQR, 31). Heartburn, regurgitations, and respiratory symptoms were fully eliminated in 46 out of 60 (77%, *p* = 0.0001), 24 out of 31 (78%, *p* = 0.0001), and 17 out of 22 (77%, *p* = 0.0001) of the patients and improved in 56 out of 60 (93%, *p* = 0.0001), 29 out of 31 (94%, *p* = 0.0001), and 20 out of 22 (91%, *p* = 0.0001) patients, respectively. A graphic comparison of the three most reported symptoms before and after MSA is shown in Fig. 2. Only 7 out of 67 (10%, *p* = 0.0001) patients reported a need for use of PPIs postoperatively.

### Postoperative Adverse Effects

**Fig. 3:**
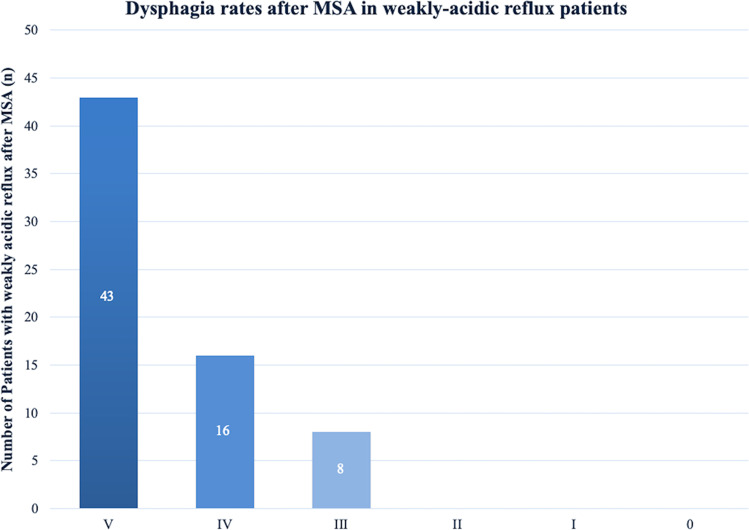
Frequency and degree of postoperative dysphagia in weakly acidic patients based on the classification of Saeed et al. Columns from up to down: **0** = Unable to swallow (0). **I** = Swallowing liquids with difficulty, solids impossible (0). **II** = Swallowing liquids without difficulty, solids impossible (0). **III** = Occasionally difficulty swallowing with solids (8). **IV** = Rarely difficulty swallowing with solids (16). **V** = Swallowing normally (43)

After MSA, a total of 43 out of 67 (64%) individuals reported absolutely no difficulty swallowing with solids or liquids. Rarely difficulties swallowing with solids only was reported by 16 out of 67 (24%) the patients, while 8 out of 67 (12%) patients had occasional difficulties swallowing with solids. Finally permanent dysphagia, defined as not being able to swallow solids or/and liquids, was not seen in any of the patients at time of follow-up. A graphic depiction of postoperative dysphagia is shown in Fig. 3.

A total of 55 out of 67 (82%) of the patients retained their ability to belch/vomit and only 5 out of 67 (8%) complained about increased daily gas bloating.

None of the individuals needed revision surgery or balloon dilatations. No erosion or migration of the device was seen. Postoperative outcomes are shown in Table 2.

**Table 2:** Comparison of postoperative outcome measures in weakly acidic reflux vs. acidic reflux patients after magnetic sphincter augmentation (MSA)

	Weakly acidic reflux patients	Acidic reflux patients	
Total *n* = 268 (100%)	*n* = 67 (25%)	*n* = 201 (75%)	
Persistent dysphagia	0	0	
Endoscopic dilatation	0	3 (1.5%)	*p* = 0.574
Reoperation	0	6 (3%)	*p* = 0.198
Device removal	0	2 (1%)	
Re-hiatoplasty	0	4 (2%)	
Gas bloating syndrom	5 (7%)	11 (6%)	*p* = 0.556
Ability to belch/vomit	55 (82%)	167 (83%)	*p* = 0.727
Median total GERD-HRQL score	2 (5)	2 (4)	*p* = 0.787
Median AS	9 (2)	9 (2)	*p* = 0.855
Use of PPIs	7 (10%)	18 (9%)	*p* = 0.808

Data were obtained and statistics applied, as described in “Methods” Abbreviations:* AS* alimentary satisfaction

### Quality of Life

Prior to surgery, 31 out of 67 (46%) patients had completed the GERD-HRQL score. The preoperative median GERD-HRQL score was 20 (IQR, 16, range 5–36). MSA led to a significant reduction of the GERD-HRQL total score (2 vs. 20, *p* = 0.001). Moreover, the median alimentary satisfaction (AS) of the 67 patients was rated 9 (IQR, 2). When asked if they would be willing to undergo the same surgery, in the same circumstances, 53 out of 67 (80%) patients said yes. Quality-of-Life results are presented in Table 2.

### Acidic Reflux Patients (*n* = 201)

The median age was 51 years (IQR, 21, range 19 – 81) and the median preoperative BMI was 25.6 (IQR, 5.5). Further demographics and preoperative findings are also shown in Table 1.

### Preoperative Symptoms

The three most common typical and atypical GERD-associated preoperative symptoms in our patients were heartburn (*n* = 181/201, 90%), regurgitations (*n* = 107/201, 53%), and respiratory symptoms (*n* = 75/201, 37%). A total of 175 out of 201 (87%) patients reported the use of PPIs prior to surgery.

### Surgery

The median OR time was 30 min (range, 10 – 95). The surgical approach was laparoscopic in all patients. No perioperative complications were seen. The median MSA device size implanted was 15 (range, 12 – 16). A total of 164 out of 201 (81%) individuals received additional crural closure. The median hospital stay was 1 day (IQR, 1).

### Postoperative Symptom Control

The median follow-up time was 22 months (IQR, 24). Heartburn, regurgitations, and chronic cough were fully eliminated in 148 out of 181 (81%, *p* = 0.0001), 88 out of 107 (82%, *p* = 0.0001), and 61out of 75 (81%, *p* = 0.0001) patients and improved in 172 out of 181 (95%, *p* = 0.0001), 102 out of 107 (95%, *p* = 0.0001), and 72 out of 75 (96%, *p* = 0.0001) patients, respectively. Only 18 out of 201 (9%, *p* = 0.0001) individuals reported a need for use of PPIs postoperatively.

### Postoperative Adverse Effects

After MSA, a total of 127 out of 201 (63%) patients reported absolutely no difficulty swallowing with solids or liquids. Rarely difficulties in swallowing with solids only was reported by 52 out of 201 (26%) of the patients, while 22 out of 201 (11%) patients had occasional difficulties swallowing with solids. Finally, permanent dysphagia was not seen in any of the individuals at time of follow-up.

A whole of 167 out of 201 (83%) of the patients retained their ability to belch/vomit and only 11 out of 201 (5%) complained about increased daily gas bloating.

In 3 out of 201 (1%) patients, dysphagia was successfully managed by endoscopic balloon dilatation. Six out of 201 (3%) individuals needed revision surgery: Two of the patients underwent explant of the MSA device due to unclear pain in the chest area, while the other four patients developed paraesophageal herniation of the gastric fundus and underwent re-hiatoplasty. No erosion or migration of the device was seen. Postoperative outcomes are shown in Table 2.

### Quality of Life

Prior to surgery, 102 out of 201 (51%) patients had completed the GERD-HRQL score. The preoperative median GERD-HRQL was 19 (IQR, 13, range, 2 – 39). MSA led to a significant reduction of the GERD-HRQL total score (2 vs. 29, *p* = 0.001). Also, the median alimentary satisfaction (AS) of all 201 patients was rated 9 (IQR, 2). When asked if they would be willing to undergo the same surgery, in the same circumstances, 162 out of 201 (81%) individuals said yes. Quality of life results are presented in Table 2.

### Comparison Between Weakly Acidic and Acidic Reflux

As abovementioned, we observed 67 out of 268 (25%) patients with weakly acidic reflux and 201 out of 268 (75%) patients with acidic reflux measured in the 24-h impedance-pH-metry prior to MSA. As demonstrated in Table 1, no statistically significant difference was seen between the groups other than the preoperative acid exposure.

## Discussion

MSA represents a novel surgical technique, FDA approved for the treatment of GERD in 2012.^34^ Over the years, multiple studies have demonstrated the safety and long-term effectiveness of the procedure, making it a highly standardized technique in anti-reflux surgery.^38^ Nevertheless, patients included in these studies had to adhere to strict inclusion criteria such as increased exposure to esophageal acid in the 24-h pH-metry, not having a large hiatal hernia, an esophagitis LA grade C or D, or dysphagia more than 3 times a week,^29^ thus leaving out patients with weakly acidic or non-acidic reflux. The importance of such a surgical definite GERD treatment rather than a symptomatic medical treatment lies precisely in the possible prevention of the development of long-term complications through acidic as well as non-acidic or mixed reflux. The aim of this study was to analyze the postoperative outcome of GERD patients with preoperative weakly acidic or non-acidic reflux measured in the 24-h impedance-pH-metry that underwent MSA in our highly specialized reflux center. Moreover, we compared the outcome between patients with weakly acidic reflux and those with acidic reflux.

As mentioned above, we defined weakly acidic reflux by a reduced total acidic (pH < 4) and percentage time (< 4.2%) and an increased number of total reflux episodes (> 40) in the 24-h impedance-pH-metry. This definition was based on the Lyon consensus, proposing that a total acidic (pH < 4) percentage time less than 4.2% and a total number of reflux episodes less than 40 are definitely physiological, and a total acidic (pH < 4) percentage time more than 6% and a total number of reflux episodes more than 80 are definitely pathological, while all the values in between are inconclusive. As the inconclusive values could possibly be pathological, but definitely not physiological, we chose the lower cut-off points.

As Campos et al. showed in his multivariate analyses in 1999, the three most predictive factors for a successful outcome of a Nissen fundoplication were an abnormal 24-h pH score, a typical primary symptom, and symptoms responsive to PPI therapy.^39^ We expanded our inclusion criteria to also include patients with an increased number of reflux episodes, as it can be a sign of non-acidic reflux and such patients could potentially profit from an anti-reflux operation, possibly less invasive than a Nissen fundoplication.

Regarding symptom control, our study showed promising results in patients with weakly acidic reflux: improvement of daily heartburn, regurgitations, and respiratory complaints were noted in 93% (*p* = 0.0001), 94% (*p* = 0.0001), and 91% (*p* = 0.0001) of the patients, respectively. These results are in line with the outcomes of patients with acidic reflux described: Louie et al. reported a relief of heartburn and regurgitations in 93.9% and 100%, respectively, after MSA.^40^ Furthermore, in a multicenter prospective observational study, we reported a reduction of sleep-awaking heartburn from 30.2 to 3.5%, moderate to severe regurgitations from 58.3 to 3.1%, and extraesophageal symptoms from 63.9 to 22% at 1-year follow-up.^41^ Concerning postoperative pharmacotherapy, here we show that 88% (*p* = 0.0001) of the patients did not need to use PPIs anymore, which is also in concordance with previously published articles.^17,41,42^ When comparing the improvement of the three most common symptoms (heartburn 93% vs. 95%, *p* = 0.615; regurgitations 94% vs. 95%, *p* = 0.723; and respiratory complaints 91% vs. 96%, *p* = 0.373), we found no difference between patients with preoperative weakly acidic and acidic reflux. Also, no significant difference was observed in the postoperative use of PPIs between patients with the two types of reflux (weakly acidic 7 vs. acid 18, *p* = 0.808). These findings show that patients with weakly acidic reflux also benefit from MSA, as the main goal of anti-reflux therapy is symptom control and mucosal healing.

At follow-up, 82% of our patients were able to belch/vomit (weakly acidic 55 vs. acidic 167, *p* = 0.727) and only 8% complained about daily gas bloating (weakly acidic 5 vs. acidic 11, *p* = 0.555) with no difference between our two groups. Most recently, Bonavina et al. reported a significant difference in excessive gas bloating and the ability to vomit between MSA and LF patients, specifically excessive gas bloating was reported in 10% of MSA patients, compared to 31% of LF patients, while 91% of MSA patients retained their ability to vomit if needed, compared to only 44.4% of LF patients.^42^

Persistent dysphagia, the most feared complication after anti-reflux surgery did not occur in our cohort, neither in patients with acidic nor weakly acidic reflux, at time of follow-up. However, rarely difficulties swallowing with solids was reported by 16 out of 67 (24%) patients, while 8 out of 67 (12%) patients had occasional difficulties swallowing with solids. This outcome is comparable to previous studies. Bonavina et al. reported one patient needing removal of the device due to postoperative dysphagia, while Ganz et al. found that 6% of the patients had bothersome dysphagia 5 years after MSA in a prospective study of 100 patients.^43,44^ According to current medical literature on anti-reflux surgery, patients after MSA commonly suffer from early postoperative dysphagia that resolves after 8 weeks, compared to patients after LF who later suffer from dysphagia.^38^ This can be explained by the different dietary regimes recommended postoperatively, namely a liquid/soft diet for the first 2 weeks with a gradual transition to solid foods in patients undergoing LF, compared to a solid unrestricted diet after MSA. This prevents forming of scaring tissue around the closed magnetic ring. Rarely, in 1.5–5.6% of patients, this bothersome symptom persists, and endoscopic dilatation is needed.^32,42,45^ None of our patients with weakly acidic reflux underwent endoscopic dilatation, device removal, or revisional surgery; however, the small sample size has to be taken into consideration. A safety profile analysis of the first thousand conducted MSAs showed a re-operation rate of 3.4%, all patients undergoing device removal due to dysphagia and reoccurrence of reflux symptoms.^45^ Also, a single-center cohort study, focusing on device removal after MSA, found 6.7% of patients undergoing non-emergent reoperation with removal of the MSA device, due to, most commonly, reoccurrence of heartburn or regurgitations. Furthermore, they observed two cases of full thickness erosion of the esophageal wall with partial endoluminal penetration.^46^ We found no difference in the postoperative rate of dilatation (weakly acidic 0 vs. 3, *p* = 0.574) or surgical revision (weakly acidic 0 vs. acid 6, *p* = 0.198) rate between patients with weakly acidic and acidic reflux, further showing the equally positive outcome in selected patients with a negative 24-h pH-metry.

The improved outcomes regarding symptom control are in line with the positive results regarding quality of life in our study. A significant drop in the median postoperative total GERD-HRQL score was seen in weakly acidic reflux patients after MSA (20 vs. 2, *p* = 0.001). This proves a substantial increase in quality of life. Moreover, the median alimentary satisfaction of our patients was 9, also showing an overall satisfaction in our cohort. These findings are in line with publications so far, all indicating that not only does the GERD-related quality of life improve after MSA, but overall patient satisfaction is high.^41,42,47,48^

Our study shows that patients with weakly acidic reflux, measured with a 24-h impedance-pH-metry, would profit from MSA, as this surgical technique is associated with a significant reduction in daily bothersome heartburn, regurgitations, and respiratory complaints, with favorably low morbidity and side effect rate as well as improvement in quality of life in patients. It also underlines the importance of a preoperative 24-h impedance-pH-metry where not only the total pH percentage time can be measured, as with the BRAVO capsule, but also a total number of reflux episodes as well as symptom correlation. Although the BRAVO capsule has shown to be more tolerable with comparable sensitivity to the 24-h impedance-pH-metry, reflux diagnostic centers should not fully give up on the 24-h wired catheter and patients tested negative with the wireless capsule should undergo further testing with an 24-h impedance-pH-metry.^49,50^ This method allows us to identify a high number of patients with negative total pH percentage time, but increased reflux episodes, exhibiting weakly acidic reflux, who would also potentially benefit from MSA.

Nevertheless, certain limitations of our study, such as the retrospective design, should be taken into consideration. Also, standardized objective postoperative testing with EFTs was not conducted due to the logistics of asymptomatic patients undergoing invasive diagnostic testing. Further prospective studies with larger sample sizes, as well as objective postoperative testing, would be of great value for further research.

## Conclusion

MSA leads to a significant reduction in daily, bothersome GERD symptoms, with low postoperative morbidity and increase in GERD-related quality of life, as well as alimentary satisfaction in patients with weakly acidic reflux. Furthermore, the importance of a preoperative 24-h impedance-pH-metry in detecting patients with weakly acidic reflux, who would benefit from MSA, should not be underestimated.
